# Solving the Puzzle of Metastasis: The Evolution of Cell Migration in Neoplasms

**DOI:** 10.1371/journal.pone.0017933

**Published:** 2011-04-27

**Authors:** Jun Chen, Kathleen Sprouffske, Qihong Huang, Carlo C. Maley

**Affiliations:** 1 Genomics and Computational Biology Program, School of Medicine, University of Pennsylvania, Philadelphia, Pennsylvania, United States of America; 2 Molecular and Cellular Oncogenesis, The Wistar Institute, Philadelphia, Pennsylvania, United States of America; 3 Cell and Molecular Biology Program, School of Medicine, University of Pennsylvania, Philadelphia, Pennsylvania, United States of America; 4 Helen Diller Family Comprehensive Cancer Center and Department of Surgery, University of California San Francisco, San Francisco, California, United States of America; University of Frankfurt - University Hospital Frankfurt, Germany

## Abstract

**Background:**

Metastasis represents one of the most clinically important transitions in neoplastic progression. The evolution of metastasis is a puzzle because a metastatic clone is at a disadvantage in competition for space and resources with non-metastatic clones in the primary tumor. Metastatic clones waste some of their reproductive potential on emigrating cells with little chance of establishing metastases. We suggest that resource heterogeneity within primary tumors selects for cell migration, and that cell emigration is a by-product of that selection.

**Methods and Findings:**

We developed an agent-based model to simulate the evolution of neoplastic cell migration. We simulated the essential dynamics of neoangiogenesis and blood vessel occlusion that lead to resource heterogeneity in neoplasms. We observed the probability and speed of cell migration that evolves with changes in parameters that control the degree of spatial and temporal resource heterogeneity. Across a broad range of realistic parameter values, increasing degrees of spatial and temporal heterogeneity select for the evolution of increased cell migration and emigration.

**Conclusions:**

We showed that variability in resources within a neoplasm (e.g. oxygen and nutrients provided by angiogenesis) is sufficient to select for cells with high motility. These cells are also more likely to emigrate from the tumor, which is the first step in metastasis and the key to the puzzle of metastasis. Thus, we have identified a novel potential solution to the puzzle of metastasis.

## Introduction

Clinically, the evolution of metastasis is one of the most important transitions in neoplastic progression. Prior to metastasis, most neoplasms can be cured surgically, and 5-year survival rates are often above 90%. However, once a neoplasm has spread to distant sites, some form of systemic therapy is necessary, and 5-year survival rates often fall below 15% [1]. Understanding and preventing metastasis would have a dramatic impact on the management and burden of the disease.

Bernards and Weinberg focused attention on a paradox in our understanding of the evolution of metastasis [2]. Within a neoplasm, cells compete for space and resources. (Epi)genetic instability generates new mutant clones, and those with a survival or reproductive advantage tend to spread within the neoplasm [3]. If a cell acquires a mutation that increases the chances that its offspring will emigrate from the neoplasm, that clone should be at a disadvantage within the primary neoplasm, because some of its reproductive potential is lost to emigration [4]. Clones that do not emigrate will have a net growth advantage over the emigrating clone, which should be quickly driven extinct [4], [5]. However, evidence suggests that 10^6^–10^7^ cells emigrate from a neoplasm every day yet rarely establish a growing metastasis in a new location in the body [6]. Thus the evolution of cell emigration from the primary neoplasm does not seem to be a rate limiting step in metastasis. How could a metastatic clone ever grow large enough to produce the millions of emigrating cells necessary to overcome the low probability of establishing a metastasis?

Four possible, non-mutually exclusive, solutions for the puzzle of metastasis have been proposed previously. First, a mutation that provides the potential to metastasize might have other effects (pleiotropy) that increases the survival or reproductive potential of the clone and so compensates for the fitness penalty of cell emigration [2], [5]. In a theoretical exploration of the first solution, Dingli and colleagues [4] suggested a second solution: there may be so many cells in a neoplasm that millions of *de novo* metastatic mutants may be produced every cell generation. Even if each metastatic clone is at a competitive disadvantage and tends to go extinct, new metastatic clones may continually replace them. Third, the potential to metastasize might only be triggered late in progression, by a change in the tumor microenvironment [7], allowing the clone to expand, without the fitness penalty of emigration, before the change in the microenvironment. Fourth, an early mutation might confer the potential to metastasize, but that potential may only be activated by a later mutation [2]. However, this is not actually a solution because the later mutation leads to a fitness disadvantage for the metastatic clone and that clone with both mutations should not expand, which mirrors the original framing of the problem.

Recently, we identified a fifth alternative based on dispersal theory in ecology [3], the “resource heterogeneity” solution. Dispersal theory predicts that resource heterogeneity in both space and time selects for migration in organisms [8] because organisms that move to locate regions with more resources than their current location will leave more offspring than sedentary organisms. We apply dispersal theory [8] to cancer to solve the paradox of the evolution of metastasis. There is microenvironmental variability in neoplasms - regions within a neoplasm can become transiently hypoxic [9]–[13] due to poorly regulated angiogenesis, changes in the vascular architecture and temporary occlusion or interruption of blood flow by neoplastic cells [13]–[15]. Thus, we propose that resource heterogeneity within neoplasms selects for cell “migration” - or motility - within the neoplasm, and that cell emigration from the neoplasm - or invasion - is a by-product of that selection.

The puzzle of metastasis was criticized for not being framed quantitatively [16]. Here we show that a quantitative model can illustrate a solution to the paradox of the evolution of cell emigration. Our computational model extends previous models [4], [5], [17] by including spatial effects, the dynamics of resources in that space and the evolution of the migratory phenotype. We observe the evolution of cell migration and emigration in a neoplasm under different degrees of temporal and spatial heterogeneity of resources.

## Methods

### Model Overview

We implemented an agent-based model of a neoplasm (NetLogo 4.0.2 [18], source code available upon request from the corresponding author). The neoplasm is represented as a grid of patches that store nutrients delivered by blood vessels, while cells are represented as motile agents that consume nutrients stored in the patches. For simplicity, the resources in the model are described as oxygen, though they could alternatively represent glucose or any other diffusible factor delivered through the vasculature.

Time is divided into short intervals of 12 hours. During each time step, blood vessels can form, be occluded by neoplastic cells proliferating in the confined space [15], and produce nutrients, which then diffuse. Cells can consume resources, move, reproduce, and die. See Figure S1 for a flow chart of a time step and Table S1 for model parameters and their normalized values.

### Model Behavior

*C* cells and *V* blood vessels are positioned in continuous 2D space atop a square grid of *P* patches. For each time step, every patch containing a blood vessel receives *r_i_* units of oxygen. To approximate continuous oxygen dynamics, we performed oxygen updates *t_a_* times in a time step (Eq. 1). Resource concentrations 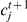 at patch 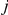 at time t+1 can be described by the following difference equation: 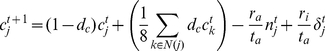
(1)where there are 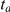 resource updates per time step in the model, *d_c_* is the resource diffusion constant, 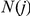 are the eight adjacent neighbour patches of 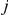, *r_a_* is the cell absorption rate, 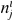 is the number of cells at position 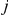 at time 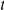, *r_i_* is the resource production rate for a microvessel and 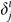 takes value 1 if there is a microvessel at position 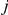 at time 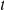 and 0 elsewhere. Resource concentrations cannot become negative because cells are prevented from absorbing more resources than are present in the location.

Our results are robust with respect to the granularity of the diffusion dynamics (*t_a_* = 100, Figure S2). In each update, *r_i_*/*t_a_* units of oxygen immediately diffuse throughout the grid by using NetLogo's discrete space diffusion function, where each patch distributes a fraction (*d_c_*) of its oxygen to its eight neighboring patches each iteration. Each cell *c_i_* consumes and stores as much oxygen as is available to it from its host patch *p_j_*, up to *r_a_/t_a_* units. Next, each cell uses *r_m_/t_a_* units of its stored oxygen *n_t,i_* for its metabolism, leaving it with *n_t,i_ - r_m_/t_a_* units. When we changed the number of blood vessels, we adjusted *r_i_* so that the total input of oxygen to the system remained constant. The parameters of oxygen dynamics were set so as to achieve realistic oxygen gradients around microvessels in normal tissues [19] (see Table S1).

Any blood vessel *v_i_* with more than *t_o_* cells in its patch is occluded and removed from the simulation. If the total number of blood vessels *v* is less than the equilibrium number *V*, then *V - v* new vessels are added randomly to hypoxic patches with less than *t_h_* units of oxygen and at least one cell, which is required to signal for angiogenesis. Angiogenesis is a complex process, including the sensing of hypoxic conditions, release of angiogenic and anti-angiogenic factors as well as endothelial cell response. The result of these processes, to a first approximation, is that new blood vessels grow into areas of hypoxic cells. Because angiogenesis is not the focus of our model, we have abstracted away most of the complexities of the process and simply maintain a homeostatic density of blood vessels, growing new blood vessels in locations where there are hypoxic cells that would release angiogenic factors.

Next, each cell *c_i_* may die (if *n_t,i_* = 0), reproduce (if *n_t,i_*>*n_r_*), or move (if 0<*n_t,i_≤n_r_*,). When a cell divides it splits its stored resources equally between its two daughter cells. During division, in both daughter cells, the migration propensity *p_i_* and maximum migration distance *m_i_* are mutated with probability *µ* by drawing a random number from a truncated normal distribution with mean *p_i_* or *m_i_* and standard deviation *sd_p_* or *sd_m_*, respectively. A cell moves with probability *p_i_*. Migrating cells move up to *m_i_* patches. These *m_i_* steps can be taken either randomly (“random migration”) or by ascending the local oxygen gradient (“gradient ascent”) as both strategies have been observed in neoplasms [20]; in both cases, the cell can move to any one of its nine closest patches (its current patch and eight neighboring patches). If during its movement it reaches a patch on the edge of the neoplasm, the cell is removed from the population and recorded as an emigrating cell.

### Model Analysis

The “variable lifespan blood vessel” model as described results in spatial and temporal resource heterogeneity because the blood vessel lifespan varies as a result of local cell dynamics. To tease apart the role of spatial and temporal effects in the variable lifespan blood vessel model, we developed a “fixed lifespan blood vessel” model in which we controlled more precisely spatial and temporal dynamics. In this model, blood vessels are destroyed after a fixed number of time steps *t_f_* rather than being occluded by cell crowding. While the fixed lifespan blood vessel model is not biologically realistic, it allows us to interpret the results from the more realistic variable lifespan blood vessel model. “Static blood vessels” can be simulated by setting the blood vessel lifespan to be infinity, and “uniform oxygen input” can be simulated by creating exactly one, static blood vessel per patch. Using this simplified model, we can control the degree of spatial heterogeneity by controlling the number of blood vessels, and the degree of temporal heterogeneity by varying the lifespan of a blood vessel (or the density of cells that cause occlusion *t_o_* in the variable lifespan blood vessel model).

Because the cells evolve to exploit the available resources, there is not a precise mapping of parameter values to available resource heterogeneity in the model. We predicted that it is the amount of available resources that is relevant to the evolution of migration. Since cells are quickly selected to utilize all available resources, only the combination of spatial and temporal heterogeneity of blood vessels in the neoplasm produces transient unutilized resources. To test this, we measured the average amount of available resource per patch, over the last 200 time steps of each model run, and evaluated its relationship to the evolution of cell migration.

We ran the model for 5,000 time steps, ∼7 simulated years, to approximate the time required to develop metastasis [21], [22]. Every parameter configuration was replicated 10 times. Data were collected and averaged over the final 200 time steps of each run. In each case, we measured the: (1) Mean migration propensity of all the cells (*p_i_*), (2) Mean maximum migration distance (*m_i_*) of all the cells in the neoplasm, and (3) Mean number of cells leaving the edge of the neoplasm (emigrating cells) per time step. We also computed the product of the first two parameters, and refer to it as the “expected migration distance” of the neoplasm, which reflects the expected distance a cell will travel in one time step. If not specified explicitly, all following experiments were done under the random migration strategy.

### Statistical Analysis

A t-test was used to test the difference of the mean equilibrium values between two simulation conditions. Linear regressions were used to quantify associations between experimentally manipulated variables (blood vessel number and lifespan) or their outcomes (observed degree of resource heterogeneity) with the expected migration distance and number of migratory cells.

## Results

Each of the model variants and parameter conditions corresponds to angiogenesis and cell movement behaviors within a neoplasm. For each of the experiments that follow, we observe the evolution of cell migration and emigration from a neoplasm across the range of parameters.

Because the amount of cell movement within a neoplasm early in progression is unknown, we tested several reasonable initial conditions within which a migratory cell could evolve: (1) Cells are generally stable and do not move, (2) Cells have a low level of movement within a neoplasm, and (3) Each cell within a neoplasm can have a different level of motility. We tested these three initial conditions by initializing (1) All cells with a migration propensity of 0 and a maximum migration distance of 0; (2) All cells with a migration propensity of 0.05 and a maximum migration distance of 1; and (3) Cells with random migration propensity values and maximum migration distance values with uniform probability over the intervals [0, 0.6] and [0, 6] respectively. We found that the initial amount of cell motility had no influence on the evolution of the final levels for both phenotypes (Figure S3; t-test *P*>0.05 for all outcomes).

**Figure 1:**
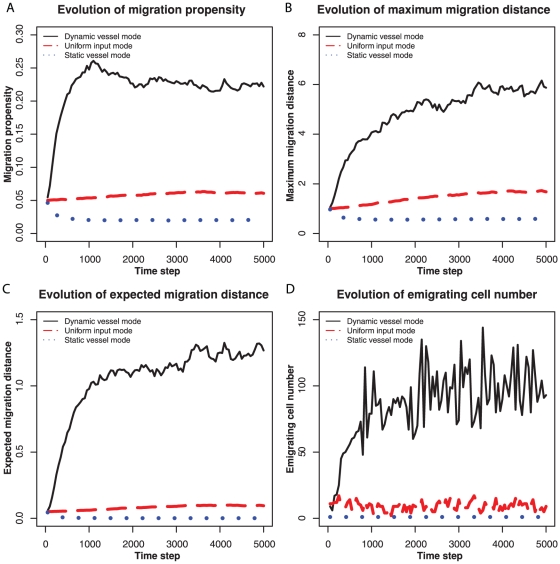
Examples of evolution of migration with dynamic blood vessels, uniform input, and static blood vessels. (A) Evolution of the migration propensity. (B) Evolution of the maximum migration distance per time step. (C) Evolution of the expected migration distance (the product of migration propensity and maximum migration distance). (D) The number of migratory cells leaving the neoplasm per time step. In the static blood vessel model (dotted lines), 100 blood vessels remain fixed in position throughout the run of the model. In the dynamic model with variable lifespan blood vessels (solid lines), a blood vessel was occluded when there were more than 20 cells in its location (patch) and replaced by a new blood vessel in a hypoxic location. In the uniform input model (dashed lines), all patches received an equal amount of resources each time step.

**Figure 2:**
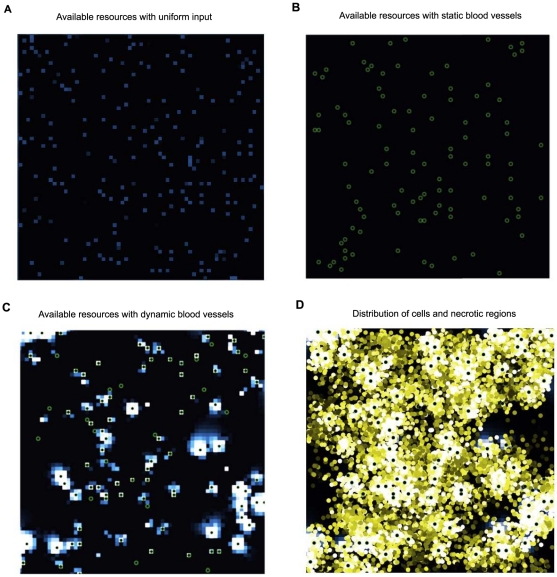
Resource and cell densities in the model. Green circles show the position of blood vessels and resource density is represented on a continuum from blue (low) to white (high). When resources flow into the tissue uniformly (A) or through static blood vessels (B), the cells consume all of the resources and the spatial heterogeneity of unutilized resources is low. When blood vessels are dynamic due to occlusion and angiogenesis (C), heterogeneity of available resources is greater because there is a lag time between the appearance of a new blood vessel and increased density of cells in that locale. This explains the differences in the evolution of cell migration for the different resource input modes shown in Figure 1. When resources flow into a tissue through sparse blood vessels, patches of normoxia and hypoxia lead to corresponding regions of high densities of cells as well as necrotic regions. Panel D shows an overlay of the cell density for the blood vessels and resources of panel C. The brightness of the cells in panel D represents the amount of resources each cell has accumulated.

We then compared the evolution of migration under the three oxygen input methods: “uniform resource input”, “static blood vessels”, and “variable lifespan blood vessels.” In the uniform resource input simulations, each patch received a constant amount of oxygen input every time step, which resembles the resource input in normal, adequately oxygenated tissue. In the static and variable lifespan blood vessel simulations, resources were distributed via a constant number of blood vessels, which either stayed in the same locations for the entire run or changed their locations. The variable lifespan blood vessel model simulates the blood vessel dynamics observed in a neoplasm. Comparison of the static blood vessel model to the variable lifespan blood vessel model allows us to test the effects of temporal heterogeneity on the evolution of cell motility. A variable lifespan blood vessel was occluded when more than 20 cells occupied its patch and was replaced by a new vessel in a hypoxic patch. The value of this parameter did not affect the qualitative results. Neoplasms with variable lifespan blood vessels evolved higher values for the migration propensity and maximum migration distance than the other two resource input models (Figure 1). The combination of spatial and temporal heterogeneity generates transient regions of unexploited resources (Figure 2). Note that static blood vessels do not produce heterogeneity of unutilized resources because cells proliferate around the blood vessels until they consume all available resources (Figure 2B). Uniform input of resources across the entire environment can lead to more available resources and greater evolution of cell migration than static blood vessels because the uniform input of resources leads to fewer cells at each source of resources and so more stochasticity of cell dynamics in each patch. The spatial heterogeneity generated by blood vessels leads to patches of necrosis and hypoxia that are typical of a neoplasm (Figure 2D).

**Figure 3:**
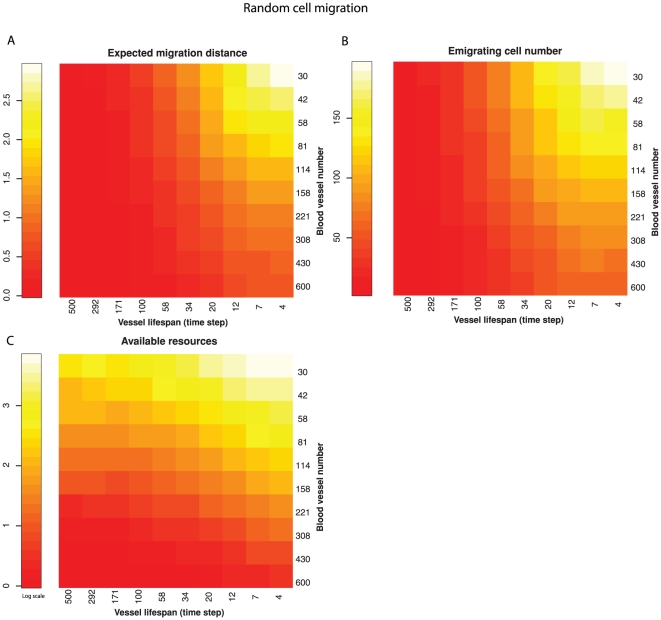
Relationship between resource heterogeneity and selection for migration when cells move randomly. Spatial heterogeneity is varied with the number of blood vessels providing resources. Temporal heterogeneity is determined by varying the lifespan of the blood vessels. As temporal and spatial heterogeneity increased the product of migration propensity and maximum migration distance (expected migration distance) increased (A) as did the number of cells emigrating from the neoplasm (B). The amount of transiently unutilized, available resources is also maximized by increasing spatial and temporal heterogeneity (C).

We ran the fixed lifespan blood vessel model with number of blood vessels from 30 to 600 and vessel lifespans from 4 to 500 time steps on a log scale, which covered the ranges observed in real neoplasms (Table S1). Decreasing blood vessel numbers and lifespan (increasing spatial and temporal heterogeneity) selected for increased cell migration and number of emigrating cells (Figure 3; linear regressions *P*<0.001). The emigrating cell number and expected migration distance are closely correlated (r  = 0.984, *P*<0.001).

**Figure 4:**
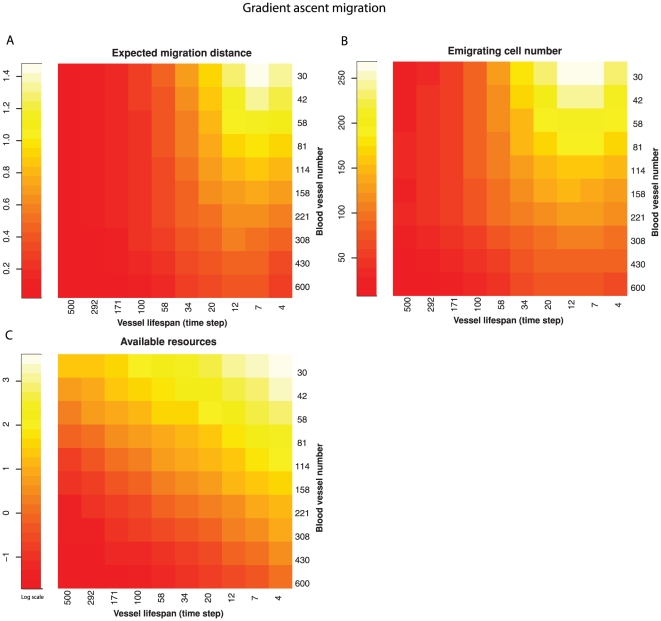
Relationship between resource heterogeneity and selection for migration when cells move up resource gradients. Spatial heterogeneity was controlled by varying the number of blood vessels and temporal heterogeneity was controlled by varying their lifespan. As with random movement, increasing temporal and spatial heterogeneity both select for increasing cell migration (expected migration distance); A) and cell emigration from the neoplasm (B). The combination of both spatial and temporal heterogeneity leads to increased amounts of transiently unutilized, available resources (C).

We repeated the experiments under the condition in which cells climb up resource gradients [20] (Figure 4). If a cell reached a local maximum of resources, it remained there even if it had the capacity to move further. Here, resource heterogeneity still selects for cell migration with the similar pattern as in neoplasms using random migration strategy (Figure 4). The association between resource heterogeneity (both temporal and spatial) and the expected migration distance remains strong (linear regression *P*<0.001), as does their relationship with the number of cells that migrate off the edge of the neoplasm (linear regression *P*<0.001). In a real neoplasm, resource concentrations may increase at the borders of the neoplasm (and other routes of exit) and so migratory cells following those gradients may exit the neoplasm more frequently than we have represented in our model.

**Figure 5:**
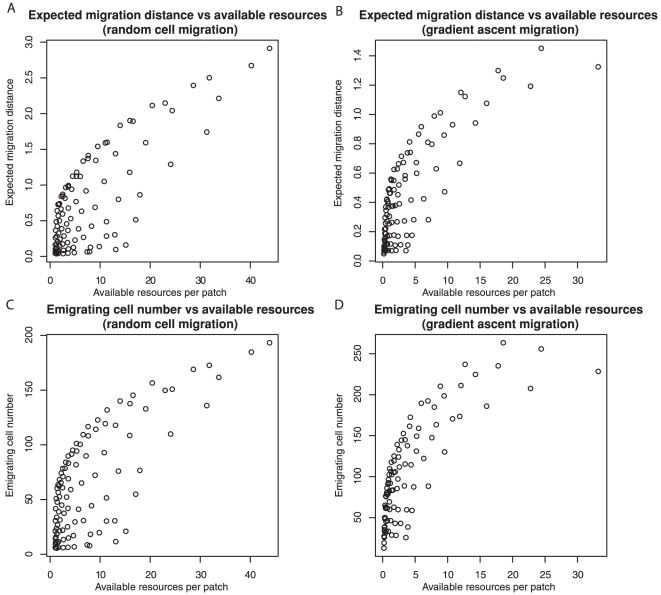
Available resources, those not currently being fully exploited by the cell population, select for increased cell migration. The expected migration distance that evolved (A, B) was closely correlated with the average amount of transiently unutilized, available resources per patch. Similarly, the number of cells emigrating from the neoplasm was also highly correlated with amount of available resources (C, D). This was true, regardless of whether cell migration was random (A, C) or by gradient ascent (B, D).

We found that the fewest number and shortest lifespan of blood vessels led to the maximal amount of available, unutilized resources, which was statistically significantly associated with the evolution of expected migration distance (Figures 5A and 5B; r = 0.79 for random migration, r = 0.81 for gradient ascent, *P*<0.001 for both), and the number of emigrating cells (Figures 5C and 5D; r = 0.74 for random migration, r = 0.79 for gradient ascent, *P*<0.001 for both). Figure S4 shows snapshots of available resources at the four extreme settings of the number of blood vessels and their lifespans.

In the model, the parameter values for the mutation standard deviation of migration propensity (*sd_p_*) and maximum migration distance (*sd_m_*) affect the rate of evolution and are set empirically. To see whether *sd_p_* or *sd_m_* affect the above results, we ran the model with different values of *sd_p_* (0.01, 0.1) and *sd_m_* (0.1, 1). Though the final evolved values for these migration phenotypes vary with different *sd_p_* or *sd_m_*, neoplasms with variable lifespan blood vessels still evolved higher values for all migration phenotypes (Figure S5).

To explore the interaction of selection for migration with selection for proliferation, we allowed cells to evolve the ability to divide with fewer resources. Increased proliferation is a hallmark of carcinogenesis [23]. When cells could optimize their proliferation under restricted resources, cell migration rates evolved to even higher levels than before. Neoplasms with variable lifespan blood vessels quickly evolved a lower threshold of resources necessary to divide (increased their proliferation rate), compared to uniform resource input or static blood vessels (Figure S6).

All the prior experiments were run on a grid of 4,096 patches. We tested neoplasms of 1024, 2116, 4096, 8100, and 16,384 patches to determine how the dynamics scale with the size of the simulated neoplasm. The fixed lifespan blood vessel model, with 6 time step lifespans, was used in these simulations. Blood vessel numbers were scaled with neoplasm size from 25, 50, 100, 200, up to 400 respectively, to ensure that the blood vessel density was the same regardless of neoplasm size. Each blood vessel delivered the same amount of resources per time step, regardless of the size of the neoplasm, so that larger neoplasms received more total resources and could support a larger population of neoplastic cells. Resource heterogeneity and expected migration distance appear to be sensitive to boundary effects in small simulations, but approach an equilibrium value (and have lower variance) in simulations of >8,000 patches (Figures S7A and S7B). The emigrating cell number scales linearly with neoplasm size, and so the frequency of cell emigration is stable over changes in neoplasm size (Figures S7A and S7C). Selection for cell migration by temporal and spatial resource heterogeneity holds regardless of the size of the simulated neoplasm.

## Discussion

Using a computational model, we explored a possible solution to the paradox of the evolution of metastasis identified by Bernards and Weinberg[2]. We propose that resource heterogeneity selects for cell motility, which leads to emigration from the primary tumor. Our model captures the fitness disadvantage associated with cell migration in that emigrating cells are removed from the model. Intriguingly, we observed that these same “disadvantageous” clones were favored in conditions predicted by dispersal theory in ecology. Namely, we have shown that spatial and temporal resource heterogeneity selects for cell migration within a neoplasm, and as a by-product, emigration from a neoplasm. Specifically, the migration propensity, the maximum migration distance within a neoplasm, and the resulting number of emigrating cells, were maximized when there were only a few blood vessels in the model and when the location of those resource rich patches changed frequently (Figures 3, 4, 5) as is thought to occur in neoplasms[9]–[12], [14], [15], [24]. Oxygen levels can fluctuate in neoplasms over a period of 10′s of minutes, in a spatially heterogeneous manner, the details of which vary between neoplasms [10], [12]. Transient hypoxia has been observed to occur over periods of minutes to hours [10] and chronic hypoxia over longer time scales [13]. Thus, our simulation results confirm that selection for migration within a neoplasm under resource heterogeneity can result in increased levels of cell emigration from the neoplasm, providing support for the resource heterogeneity solution to the paradox of the evolution of metastasis.

The resource heterogeneity solution to the paradox of metastasis is consistent with a variety of experimental observations, including spatial and temporal patterns of tumor invasion, patterns of gene expression in the primary tumor that predict metastasis, and the metastatic effects of hypoxia on neoplasms. Our model is consistent with observations of rapid metastasis once a neoplasm becomes malignant [21], because we predict that there has been selection for cell migration prior to invasion. In gene expression studies, primary neoplasms often exhibit an expression signature of metastasis [25]–[29]. Since expression arrays measure the most common clones in the neoplasm, this has been interpreted as evidence that a metastatic phenotype often evolves early in neoplastic progression [2]. These gene expression profiles may actually be a signature of resource heterogeneity or of migratory clones. For example, cell motility and stress response genes were enriched in primary neoplasms associated with recurrence [26]. Hypoxia has also been associated with increased risk of metastasis [9], [30]–[33]. The resource heterogeneity hypothesis predicts that temporal variation in hypoxia should select for increased emigration, and this is consistent with observations in mouse models [34]–[36]. Intriguingly, a molecular mechanism connects hypoxic stress and migration through HIF1-α [37], suggesting that natural selection could co-opt and optimize the (epi)genetics of cells under hypoxic stress to increase cell migration.

The paradox of the evolution of metastasis depends on the observation that emigration is a competitive disadvantage for clones in the primary tumor, and so natural selection should suppress cell emigration. The following steps in metastasis (e.g., survival in the blood, invasion and establishment in a new location, etc. [38]) are all selectively advantageous for the emigrating clone, and so are not paradoxical. We have focused here on the evolution of the first step of metastasis: migration of cells within the neoplasm, which leads to emigration from the neoplasm, or invasion in our model. The paradox of metastasis hinges on this first step.

The possible resolutions to the paradox of metastasis are distinct in our model, including that metastatic mutations may also increase fitness, the mutation rate is high enough to generate the metastatic cells *de novo*, and microenvironmental changes “activate” a previously neutral mutation late in progression. In our model, mutations only affect the propensity or speed of migration, and do not directly affect apoptosis or proliferation. *De novo* migratory mutations cannot explain the evolution of high rates of migration observed in our models. That being said, the other solutions to the puzzle of metastasis are not mutually exclusive with each other or our proposal. There is evidence that some mutations that facilitate metastasis may also increase the fitness of the mutant clone [39], [40].

Our model is clearly a simplification of intra-tumor dynamics. In a real neoplasm, cells are likely to emigrate through lymphatic and blood vessels, not just by leaving the borders of the neoplasm. Incorporating those details into our model would likely increase the number of emigrating cells, consistent with the behavior of our current model.

One of the weaknesses of models of metastasis is the lack of experimental data on cell migration and hypoxia, particularly at the single cell level *in vivo*. Thus, we have used a quantitative model to explore the puzzle of metastasis and develop a hypothesis that can explain current data and be used to guide future experiments. Our model supports previous predictions [41] that assays of spatial and/or temporal heterogeneity of available resources in a neoplasm [10] should predict the risk of metastasis. Spatial statistics of patchiness could be applied to assays of hypoxia, glucose or other limiting resources in tissue sections [9]–[13]. We also predict that direct measures of cell migration in the primary tumor, perhaps through measures of genes expression and proteins in cell migration pathways, should be good biomarkers for the risk of metastasis.

There are a number of additional experimentally testable predictions from our model. First, our model suggests that we should find greater expression of migration related proteins in neoplasms with regions of hypoxia compared to neoplasms with uniform oxygenation. If the half-lives of hypoxia inducible markers are significantly longer than the rate of cell movement, migratory cells with those markers might be detected as recent arrivals in normoxic regions. With a fast enough molecular clock, perhaps through methylation of CpG sites [42], one may be able to show more mixing of cell lineages due to migration in a neoplasm with resource heterogeneity compared to neoplasms with uniform resources which should contain contiguous regions of closely related cells.

Interestingly, results from our model of resource heterogeneity suggest a potential strategy for preventing or delaying cancer: normalizing the resources available to a neoplasm, over space or time, should tend to reduce the risk of metastasis. In fact, it has recently been shown that restoration of neoplasm oxygenation suppresses metastasis [43]. Our model also predicts that cycles of anti-angiogenic drugs applied to a pre-malignant neoplasm may select for a metastatic clone and so we should be cautious in the application of such drugs for cancer prevention [44], [45]. It has recently been shown that a decrease in tumor vascularity is correlated with tumor invasion in gliobalstoma patients treated with anti-angiogenesis therapy [46]. These results are consistent with our model results that migration increases when the vessel density is decreased. Nevertheless, there is both theoretical and experimental support for anti-angiogenic therapy in malignant neoplasms [47]. In fact, constant, low doses of anti-angiogenic drugs have been shown normalize the vascular networks within neoplasms [48]–[50]. Thus, the chronic application of such drugs may be a route to normalizing the spatial and temporal resources of a neoplasm, thus preventing selection for cell migration and metastasis.

In a related model, Bearer et al. studied the effects of resource heterogeneity and competition between a low- and high-grade clone on tumor morphology and came to a similar conclusion[51]. This model represented physical and chemical constraints, along with cell adhesion dynamics to predict how the interface between tumor and normal tissue changes over time. In this model, cell migration was a cellular response to hypoxia and did not evolve. In contrast, our model does not represent the boundary between tumor and normal tissue, and instead focuses on the selective effects of resource heterogeneity on cell migration within the primary tumor. They found that resource heterogeneity was amplified by cellular proliferation and migration, leading to invasive tumor morphologies. From this complementary approach, they also concluded that normalization of resources should help suppress invasion. In their case, because resource homogeneity leads to physical constraints on tumor shape whereas in our case, resource homogeneity suppresses natural selection for cell motility.

We have provided a quantitative model for the evolution of cell migration and emigration from neoplasms that provides a solution to the puzzle of metastasis. Results from the model are consistent with both expression signatures of metastasis in primary neoplasms [25]–[29] and the observed association between hypoxia and metastasis [9], [30]–[32]. We propose that cell emigration from a neoplasm is a side effect of selection for migration within a neoplasm. The results of our model do not rely upon the exact details of the model. Regardless of the precise parameters chosen, the result still holds that resource heterogeneity in space and time select for cell migration (see Figures S2 through S7). The predictions of our model are supported by *in vivo* experiments [34]–[36] and clinical results [9], [30]–[32]. We hope that an understanding of the evolutionary forces that select for metastasis will be useful for the future prevention of metastasis.

## Supporting Information

Figure S1 **A flow chart outlining the decisions made by cells and blood vessels for each time step.** The labeled parameters are defined in Table S1. Actions made by cells are ovals, actions made by vessels are parallelograms, and decision points are diamonds.
(PDF)

Figure S2 **Scaling the granularity of the resource dynamics (input, diffusion, cell uptake, and cell metabolism) had no effect on the results that temporal and spatial heterogeneity select for increased cell migration.** Here, instead of 10 resource dynamic iterations per cell time step, we used 100 resource iterations (t_a_). The expected migration distance (A), emigrating cell number (B) and transiently unutilized, available resources (C) are all strongly affected by both spatial and temporal heterogeneity of the resource inputs (blood vessel number and lifespan). The strong correlation between available resources and both expected migration distance (D) and the number of cells that leave the neoplasm (E) remains the same as well.
(TIF)

Figure S3 **Effect of the initial migration propensity and the maximum migration distance phenotypes on the evolution of migration in example runs of the model.** The random initial phenotypes (blue dotted lines show the population average) initialize each cell with a migration propensity randomly chosen from 0 to 0.6 and a maximum migration distance randomly chosen from 0 to 6, with uniform probability. All cells in the uniform initial phenotypes case (red lines show the population average) were initialized with a migration propensity of 0.05 and maximum migration distance of 1 patch. In all 4 panels, the neoplasm evolves to approximately the same value indicating initial phenotypes have no impact on the outcome of the model (t-test across runs of the average values over the last 200 time steps, p>0.05). (A) The evolution of the migration propensity, (B) the evolution of the maximum migration distance per time step, (C) the evolution of the expected migration distance, and (D) the evolution of the number of migratory cells leaving the neoplasm per time step is unaffected by the initial parameter settings. Here, the dynamic model with variable lifespan blood vessels used 100 blood vessels and an occlusion threshold of 20.
(TIF)

Figure S4 **The relationship between blood vessel dynamics and available resources.** In all 4 panels, the background represents the amount of available resources. White indicates that there are a lot of available resources, black that there are none, and blue that there is some small amount. Yellow circles represent the location of blood vessels. (A) When there are few blood vessels, but they have long lifespans (e.g. 500 time steps), natural selection leads to almost complete utilization of input resources. In this case, only a single recently generated blood vessel has not yet been completely exploited by the cells. (B) With few blood vessels that are generated and occluded frequently, the cells do not have enough time to locate and proliferate around a blood vessel before it disappears. This leads to large quantities of available resources for any cell that migrates from its current position, and so there is selection for increased cell migration. (C) and (D) With a high density of blood vessels, cells are distributed relatively evenly across space, though at low density for any one patch, and new blood vessels will likely appear in regions already occupied by cells that are supported by nearby blood vessels. This leaves little room for the generation of unutilized resources, though there are occasional small regions of resources generated when those blood vessels have short lifespans (D).
(TIF)

Figure S5 **Examples of the evolution of the expected migration distance under different rates of evolution determined by varying the migration propensity mutation standard deviation and the maximum migration distance mutation standard deviation.** Dynamic model with variable lifespan blood vessels (black line) selects for higher levels of cell migration in all cases, compared to uniform input of resources across space and time (red dashed line), or static blood vessels (blue dotted line). Changing the standard deviation of the daughter cell migration propensity by a factor of 10 (the “size” of mutations) has little effect on the expected migration distance, except in the slope of the initial trajectory in the variable lifespan blood vessel condition (A, B). However, the standard deviation of the maximum migration mutation does affect the expected migration distance (C, D), though, even at the low rate (std.dev.  = 0.1; panel C), the expected migration distance continues to increase and does not reach equilibrium by the end of the 7 simulated years.
(TIF)

Figure S6 **Evolution of both migration rate and the amount of resources required to reproduce.** The plots show sample runs of evolution of migration within neoplasms with non-uniform cellular proliferation rate under dynamic model with variable lifespan blood vessels, the uniform input, and the static blood vessel models. (A**)** Evolution of the migration propensity. (B**)** Evolution of the maximum migration distance per time step. (C) Evolution of the expected migration distance. (D**)** The number of migratory cells leaving the neoplasm per time step. Each panel shows the results of three different forms of resource supply to the neoplasm. In this setting, the reproduction threshold is a mutable phenotype. If a cell gets a new mutation (mutation rate  = 10^−2^ per cell division), the reproduction threshold of each daughter cell is modified by drawing from a truncated normal distribution with the parental threshold as mean and a standard deviation of 20 units. The initial reproduction threshold is set to be 240 units and the lowest threshold is 120. The other model parameters remain the same as in the uniform proliferation rate setting (Figure 1). For all four outcomes (panels A–D), neoplasms with dynamic blood vessels still evolve to the highest levels of cell migration with even higher values of expected migration distance and emigrating cell numbers. This demonstrates that adding more evolutionary complexity into our model does not change the fundamental results. (E**)** Neoplasms with dynamic blood vessels quickly evolved increased proliferation ability by lowering the threshold necessary to reproduce. Since daughter cells receive half of the parent's resources at cell division, and cells need to maintain an internal resource store in order to avoid cell death, there is selection against setting the reproduction threshold so low that daughter cells would be on the brink of starvation.
(TIF)

Figure S7 **Scaling the size of the simulated neoplasm in the fixed lifespan blood vessel model.** Due to computational constraints, we have simulated a relatively small neoplasm. To test how the simulation size might affect the results, we tested models with 1024, 2116, 4096, 8100, and 16,384 patches and fixed lifespan blood vessels. The blood vessel number was also scaled 1∶41 with neoplasm size (25, 50, 100, 200 and 400) and the lifespan of blood vessels was set to 6 time steps. The average amount of available, unutilized resources per patch (A) and the expected migration distance (B) appear to be approaching an asymptote. The number of emigrating cells appears to scale linearly as the neoplasm, and cell population size grows (C).
(TIF)

Table S1 **Parameters and their values used in the model.**
(PDF)
