# Supplementary material for: Solving the Puzzle of Metastasis: The Evolution of Cell Migration in Neoplasms
Source: PLoS One. 2011 Apr 27;6(4):e17933. doi: 10.1371/journal.pone.0017933 (PMC3083389; doi:10.1371/journal.pone.0017933)
Supplement: Figure S1 — A flow chart outlining the decisions made by cells and blood vessels for each time step. The labeled parameters are defined in Table S1. Actions made by cells are ovals, actions made by vessels are parallelograms, and decision points are diamonds. (PDF) [file pone.0017933.s001.pdf]

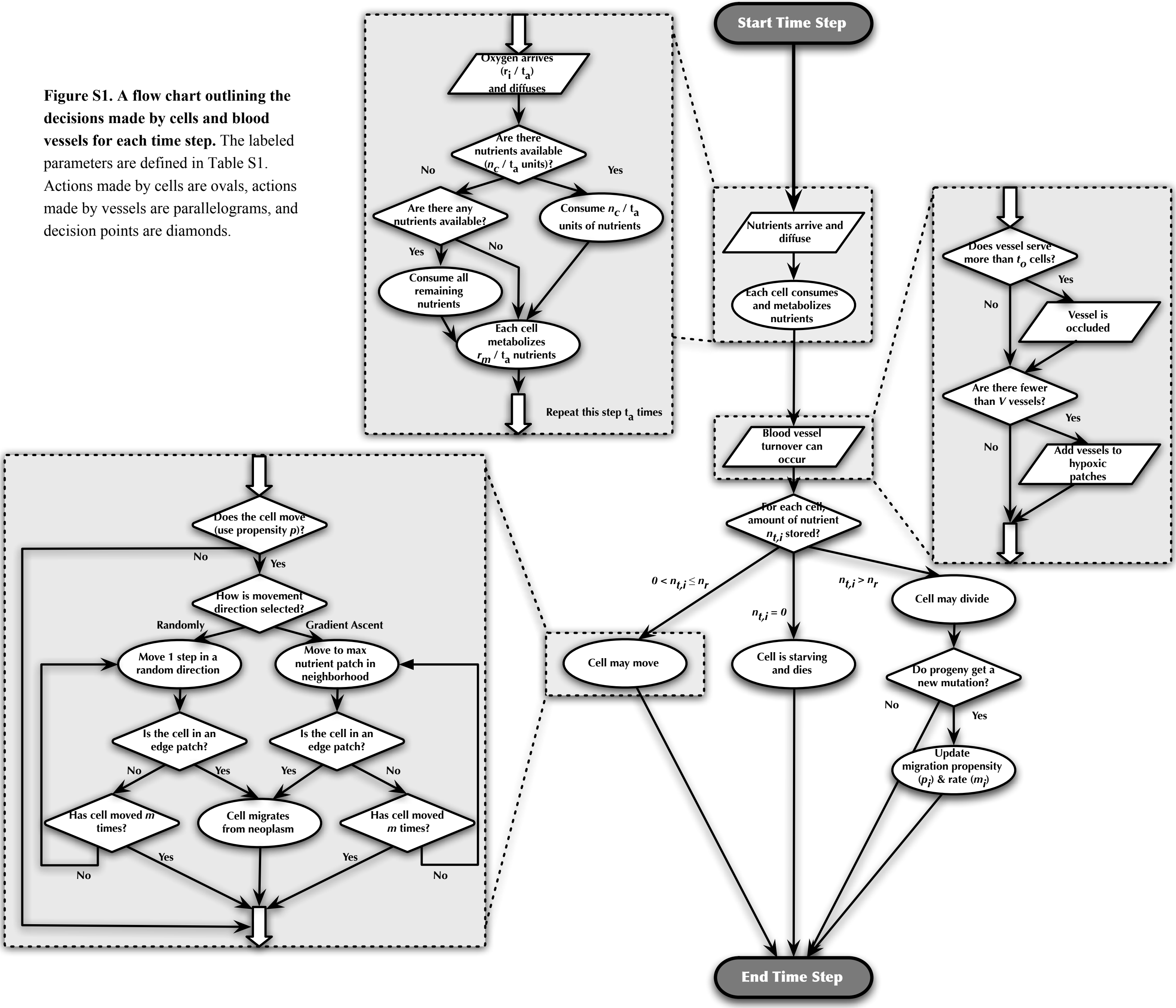

Figure S1. A flow chart outlining the decisions made by cells and blood vessels for each time step. The labeled parameters are defined in Table S1. Actions made by cells are ovals, actions made by vessels are parallelograms, and decision points are diamonds.
