# Supplementary figures and images for: Solving the Puzzle of Metastasis: The Evolution of Cell Migration in Neoplasms
Source: PLoS One. 2011 Apr 27;6(4):e17933. doi: 10.1371/journal.pone.0017933 (PMC3083389; doi:10.1371/journal.pone.0017933)

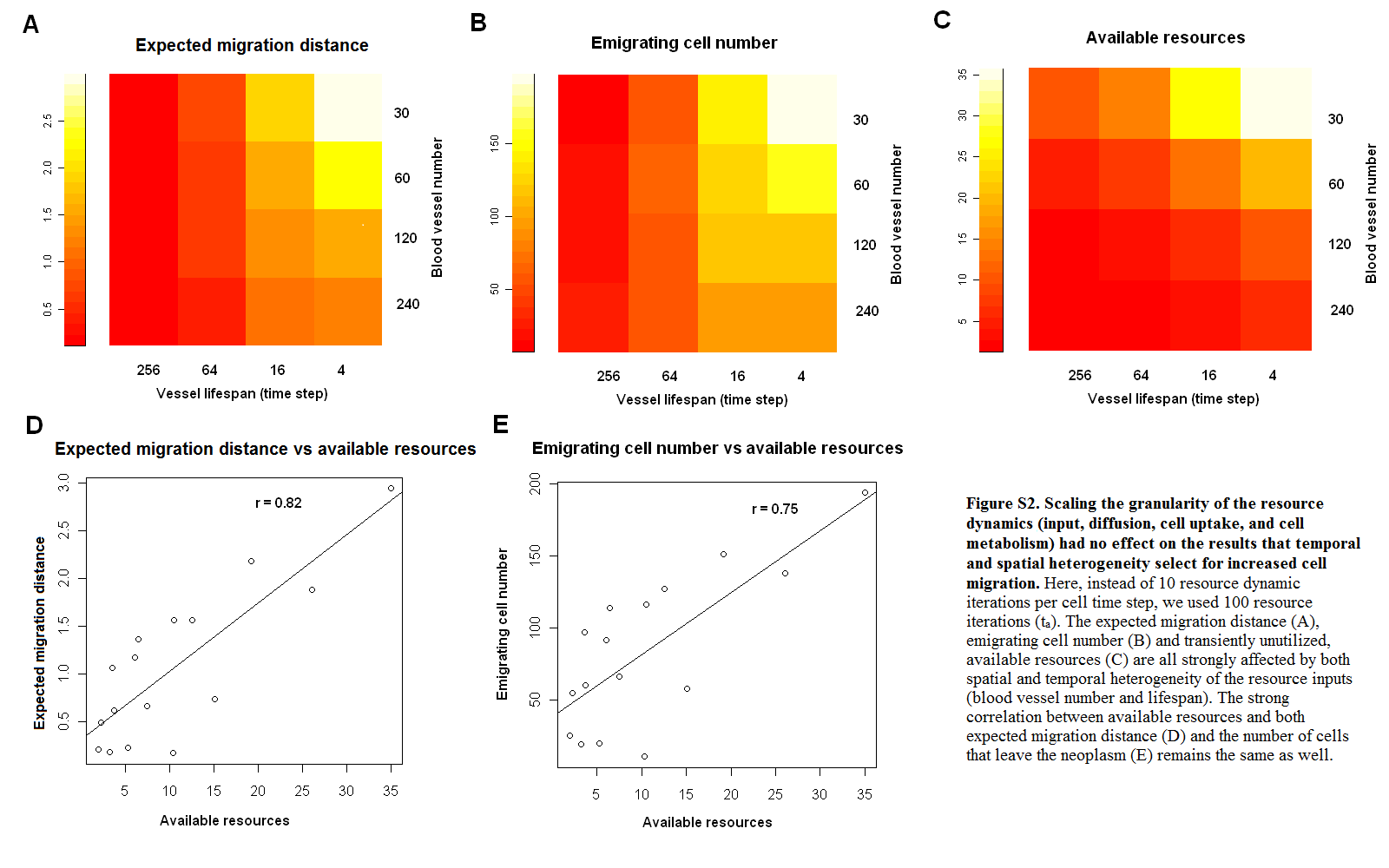

Supplement: Figure S2 — Scaling the granularity of the resource dynamics (input, diffusion, cell uptake, and cell metabolism) had no effect on the results that temporal and spatial heterogeneity select for increased cell migration. Here, instead of 10 resource dynamic iterations per cell time step, we used 100 resource iterations (ta). The expected migration distance (A), emigrating cell number (B) and transiently unutilized, available resources (C) are all strongly affected by both spatial and temporal heterogeneity of the resource inputs (blood vessel number and lifespan). The strong correlation between available resources and both expected migration distance (D) and the number of cells that leave the neoplasm (E) remains the same as well. (TIF) [file pone.0017933.s002.tif]

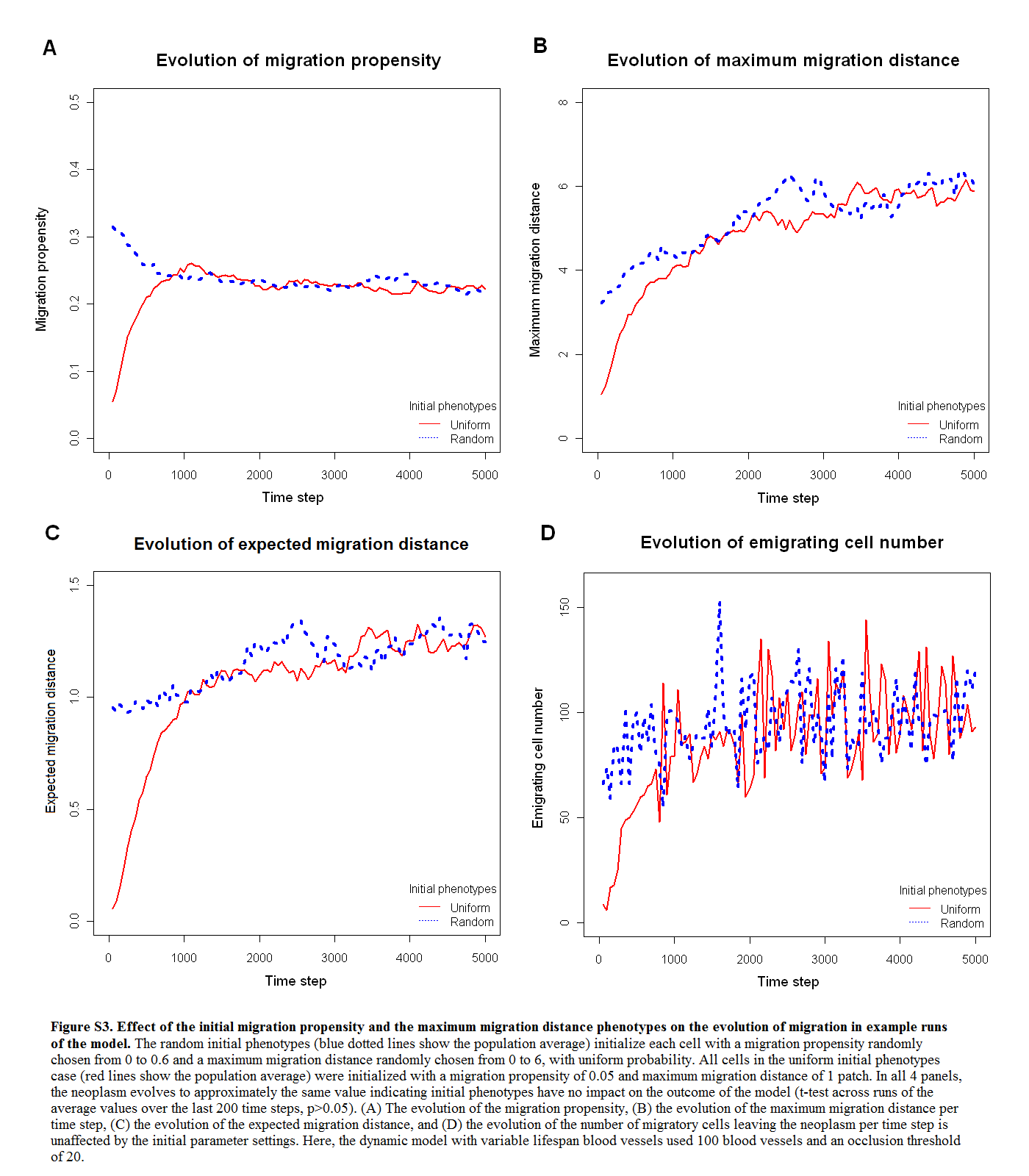

Supplement: Figure S3 — Effect of the initial migration propensity and the maximum migration distance phenotypes on the evolution of migration in example runs of the model. The random initial phenotypes (blue dotted lines show the population average) initialize each cell with a migration propensity randomly chosen from 0 to 0.6 and a maximum migration distance randomly chosen from 0 to 6, with uniform probability. All cells in the uniform initial phenotypes case (red lines show the population average) were initialized with a migration propensity of 0.05 and maximum migration distance of 1 patch. In all 4 panels, the neoplasm evolves to approximately the same value indicating initial phenotypes have no impact on the outcome of the model (t-test across runs of the average values over the last 200 time steps, p>0.05). (A) The evolution of the migration propensity, (B) the evolution of the maximum migration distance per time step, (C) the evolution of the expected migration distance, and (D) the evolution of the number of migratory cells leaving the neoplasm per time step is unaffected by the initial parameter settings. Here, the dynamic model with variable lifespan blood vessels used 100 blood vessels and an occlusion threshold of 20. (TIF) [file pone.0017933.s003.tif]

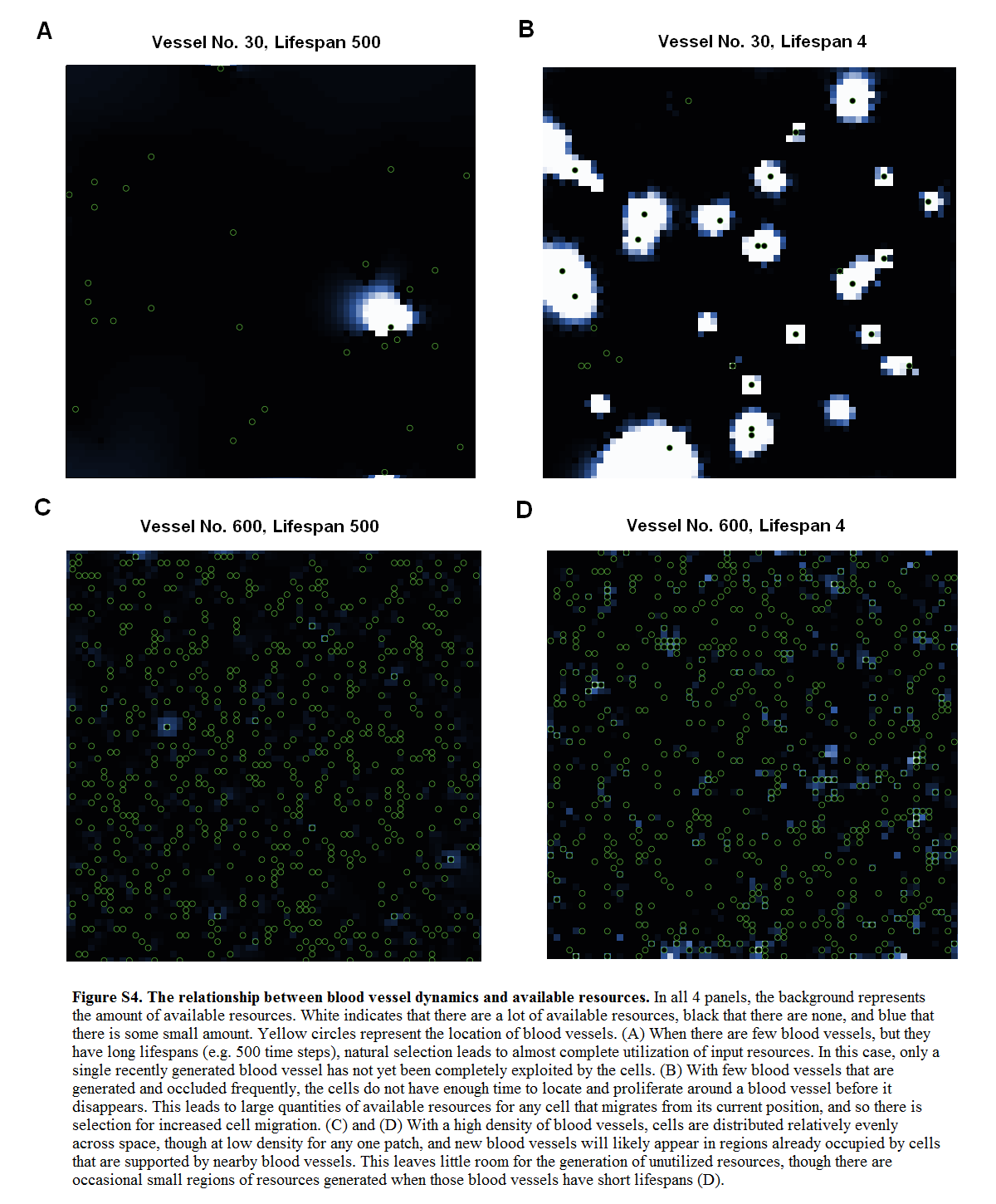

Supplement: Figure S4 — The relationship between blood vessel dynamics and available resources. In all 4 panels, the background represents the amount of available resources. White indicates that there are a lot of available resources, black that there are none, and blue that there is some small amount. Yellow circles represent the location of blood vessels. (A) When there are few blood vessels, but they have long lifespans (e.g. 500 time steps), natural selection leads to almost complete utilization of input resources. In this case, only a single recently generated blood vessel has not yet been completely exploited by the cells. (B) With few blood vessels that are generated and occluded frequently, the cells do not have enough time to locate and proliferate around a blood vessel before it disappears. This leads to large quantities of available resources for any cell that migrates from its current position, and so there is selection for increased cell migration. (C) and (D) With a high density of blood vessels, cells are distributed relatively evenly across space, though at low density for any one patch, and new blood vessels will likely appear in regions already occupied by cells that are supported by nearby blood vessels. This leaves little room for the generation of unutilized resources, though there are occasional small regions of resources generated when those blood vessels have short lifespans (D). (TIF) [file pone.0017933.s004.tif]

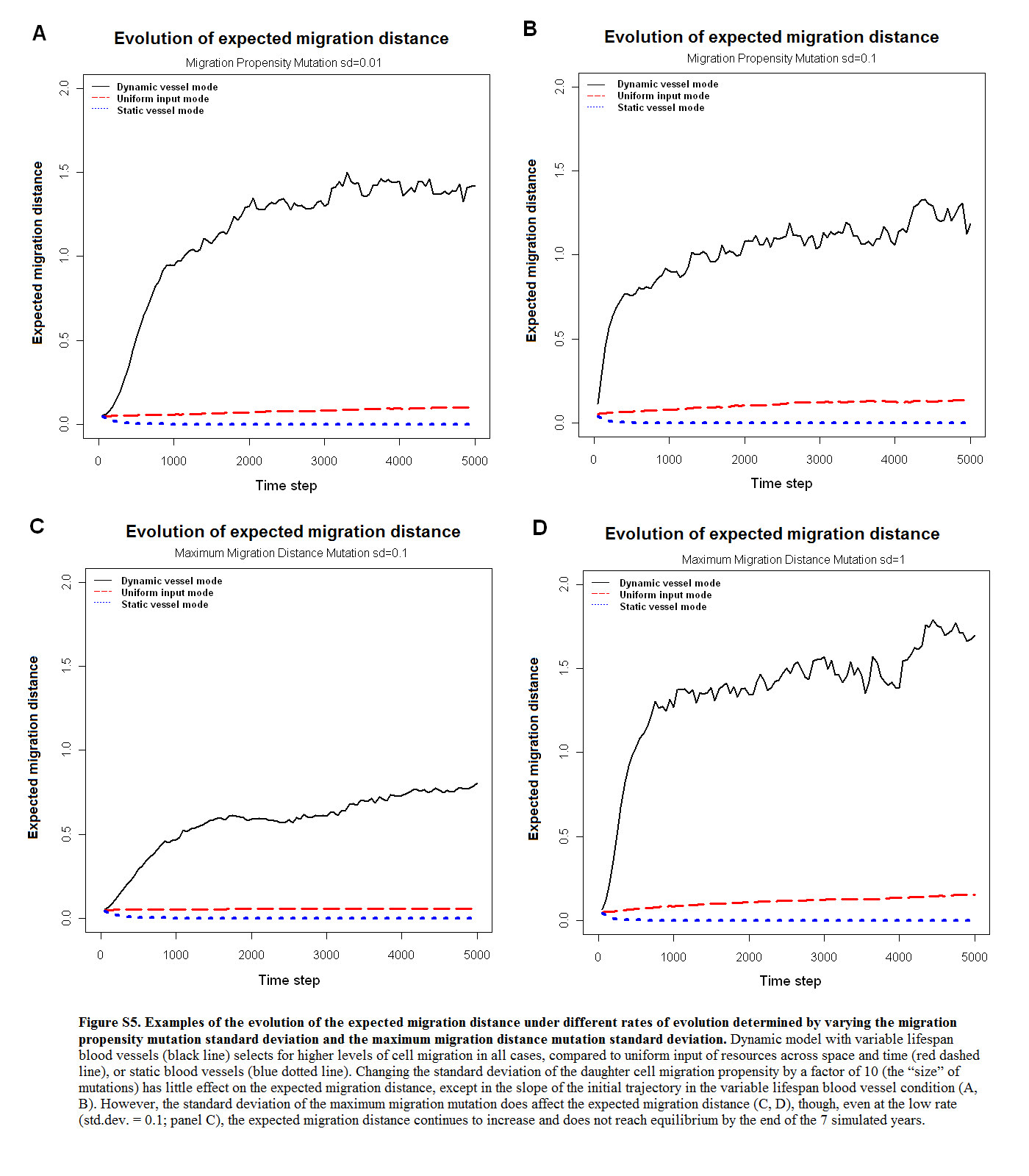

Supplement: Figure S5 — Examples of the evolution of the expected migration distance under different rates of evolution determined by varying the migration propensity mutation standard deviation and the maximum migration distance mutation standard deviation. Dynamic model with variable lifespan blood vessels (black line) selects for higher levels of cell migration in all cases, compared to uniform input of resources across space and time (red dashed line), or static blood vessels (blue dotted line). Changing the standard deviation of the daughter cell migration propensity by a factor of 10 (the “size” of mutations) has little effect on the expected migration distance, except in the slope of the initial trajectory in the variable lifespan blood vessel condition (A, B). However, the standard deviation of the maximum migration mutation does affect the expected migration distance (C, D), though, even at the low rate (std.dev. = 0.1; panel C), the expected migration distance continues to increase and does not reach equilibrium by the end of the 7 simulated years. (TIF) [file pone.0017933.s005.tif]

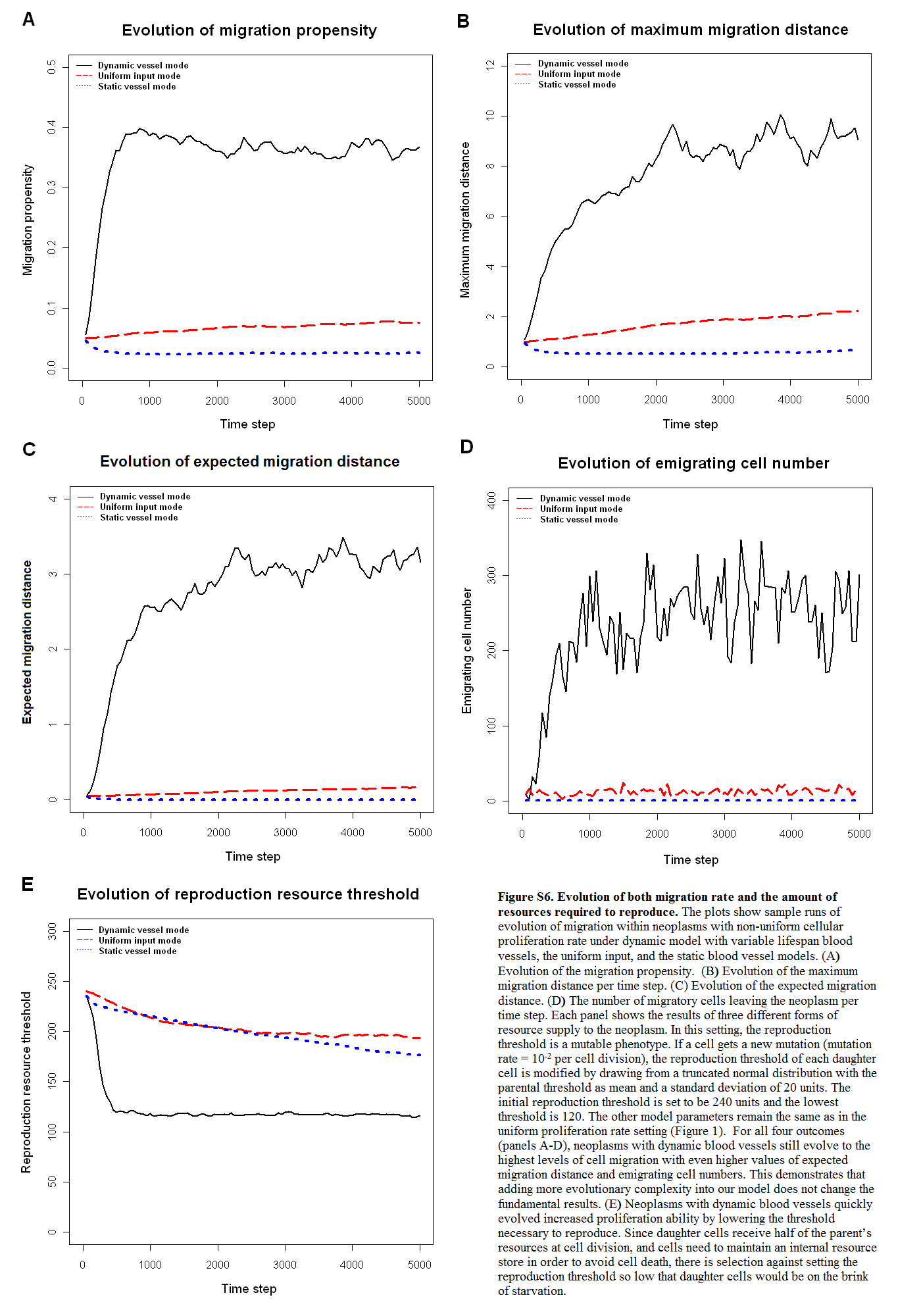

Supplement: Figure S6 — Evolution of both migration rate and the amount of resources required to reproduce. The plots show sample runs of evolution of migration within neoplasms with non-uniform cellular proliferation rate under dynamic model with variable lifespan blood vessels, the uniform input, and the static blood vessel models. (A) Evolution of the migration propensity. (B) Evolution of the maximum migration distance per time step. (C) Evolution of the expected migration distance. (D) The number of migratory cells leaving the neoplasm per time step. Each panel shows the results of three different forms of resource supply to the neoplasm. In this setting, the reproduction threshold is a mutable phenotype. If a cell gets a new mutation (mutation rate = 10−2 per cell division), the reproduction threshold of each daughter cell is modified by drawing from a truncated normal distribution with the parental threshold as mean and a standard deviation of 20 units. The initial reproduction threshold is set to be 240 units and the lowest threshold is 120. The other model parameters remain the same as in the uniform proliferation rate setting (Figure 1). For all four outcomes (panels A–D), neoplasms with dynamic blood vessels still evolve to the highest levels of cell migration with even higher values of expected migration distance and emigrating cell numbers. This demonstrates that adding more evolutionary complexity into our model does not change the fundamental results. (E) Neoplasms with dynamic blood vessels quickly evolved increased proliferation ability by lowering the threshold necessary to reproduce. Since daughter cells receive half of the parent's resources at cell division, and cells need to maintain an internal resource store in order to avoid cell death, there is selection against setting the reproduction threshold so low that daughter cells would be on the brink of starvation. (TIF) [file pone.0017933.s006.tif]

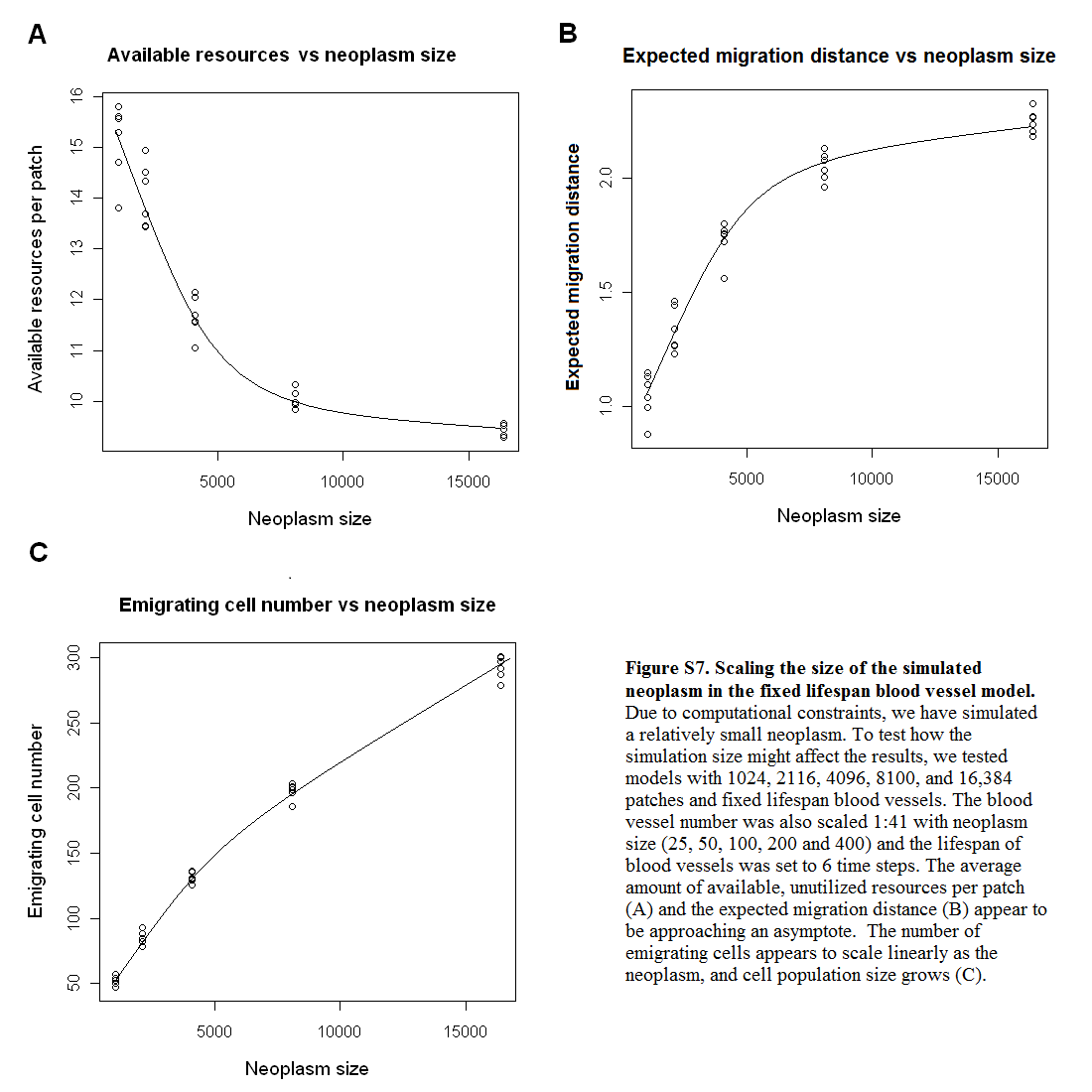

Supplement: Figure S7 — Scaling the size of the simulated neoplasm in the fixed lifespan blood vessel model. Due to computational constraints, we have simulated a relatively small neoplasm. To test how the simulation size might affect the results, we tested models with 1024, 2116, 4096, 8100, and 16,384 patches and fixed lifespan blood vessels. The blood vessel number was also scaled 1∶41 with neoplasm size (25, 50, 100, 200 and 400) and the lifespan of blood vessels was set to 6 time steps. The average amount of available, unutilized resources per patch (A) and the expected migration distance (B) appear to be approaching an asymptote. The number of emigrating cells appears to scale linearly as the neoplasm, and cell population size grows (C). (TIF) [file pone.0017933.s007.tif]
