# Supplementary material for: Solving the Puzzle of Metastasis: The Evolution of Cell Migration in Neoplasms
Source: PLoS One. 2011 Apr 27;6(4):e17933. doi: 10.1371/journal.pone.0017933 (PMC3083389; doi:10.1371/journal.pone.0017933)
Supplement: Table S1 — Parameters and their values used in the model. (PDF) [file pone.0017933.s008.pdf]

Table S1. Parameters and their values used in the model.

Parameter	Meaning	Biological Value	Normalized Value	Reference
Neoplasm Size (P)	The dimensions (in patches) of the square grid representing the neoplasm.	2mm×2mm	64×64 patches (~30 μm ×30μm/patch)	(1)
Neoplasm Cell Density (n_0)	The cell density in the neoplasm.	3.0×10^3/mm^2	3 per patch	(2)
Initial Neoplasm Cell Number (C)	The initial number of cells at the start of the model	(derived from P and n_0)	~12,000 cells	(1, 2)
Vessel Number (V)	The total number of blood vessels.	7.5 - 150/mm^2	30 – 600	(2)
Oxygen Input Rate (r_i)	The amount of oxygen input into the neoplasm each time step.	2.7×10^{-14} mol/ mm^2/s (estimated based on n_0, r_m)	60 units per patch per time step	Estimated
Oxygen Diffusion Coefficient (d_c)	The percent of total oxygen in a patch distributed to its eight neighbor patches in each iteration of diffusion function.	1.8×10^{-5}cm^2/s	50% (Calibrated)	(3)
Oxygen update time (t_a)	The number of time discretization per time step.	N/A, for approximating continuous behavior	10 per time step	Model specific
Hypoxia Threshold (t_h)	The amount of oxygen in a patch below which a cell signals for angiogenesis.	0.5 – 30% of the concentration in surrounding tissue	8 units	(4)
Vessel Occlusion Threshold (t_o)	The number of cells in the same patch as the blood vessel that occludes the blood vessel.	N/A, for achieving vessel turnover rate of 5 days	20 cells	Model specific
Fixed Vessel Lifespan (t_f)	The number of time steps that a blood vessel persists before it is removed from the model	48h – 250 days	4 – 500 time steps	(5)
Cell Oxygen Absorption Rate (r_a)	The maximum amount of oxygen one cell can absorb in a time step.	4.5×10^{-17} mol/cell/s (exponential state of growth)	100 units	(6)
Cell Metabolism Rate (r_m)	The amount of resources that is metabolized in one cell per time step.	8.9×10^{-18} mol/cell/s (plateau state of growth)	20 units	(6)
Cell Reproduction Threshold (n_r)	The amount of resource units that must be accumulated before a cell can divide.	N/A, for achieving tumor cell cycle of 1.5 day	240 units	(7)
Initial Migration Propensity (p)	The initial migration probability given to all cells at the start of the model.	5% of the cell population	5%	(8)
Initial Maximum Migration Distance (m)	The initial maximum distance that a cell can migrate given to all cells at the start of the model.	3 μm/h	1 patch per time step	(9)
Mutation Rate (μ)	The probability that a phenotype will change in a daughter cell when the parental cell divides	0.01 per cell division	0.01	(10)
Migration Propensity Mutation Standard Deviation (sd_p)	The standard deviation of the normal distribution from which the mutated migration propensity value is drawn.	NA	(0.01,0.025,0.1)	Parameter Sweep
Maximum Migration Distance Mutation Standard Deviation (sd_m)	The standard deviation of the normal distribution from which the mutated maximum migration distance value is drawn.	NA	(0.1,0.5,1)	Parameter Sweep

We adjusted the NetLogo diffusion coefficient and oxygen update time to mimic the oxygen gradient observed in normal tissues. Specifically, we modeled a slice of tissue of 8 by 8 patches with one microvessel in the center (~ 120 μm radius). We turned off the cell death, reproduction and movement. Then we adjusted the oxygen update time and oxygen diffusion coefficient so that the oxygen gradient is similar to the observation in (11). This also agrees well with the fact the diffusion distance is between 100 μm and 200 μm (12).

Bibliography:

1. Basanta D, Strand DW, Lukner RB, et al. The role of transforming growth factor-beta-mediated tumor-stroma interactions in prostate cancer progression: an integrative approach. Cancer Res 2009; 69: 7111-20.
2. Okita Y, Kinoshita M, Goto T, et al. C-11-methionine uptake correlates with tumor cell density rather than with microvessel density in glioma: A stereotactic image-histology comparison. Neuroimage 2010; 49: 2977-82.
3. Grote J, Susskind R, Vaupel P. Oxygen Diffusivity in Tumor Tissue (Ds-Carcinosarcoma) under Temperature Conditions within Range of 20-40-Degrees-C. Pflug Arch Eur J Phy 1977; 372: 37-42.
4. Brown JM, William WR. Exploiting tumour hypoxia in cancer treatment. Nat Rev Cancer 2004; 4: 437-47.
5. Engerman RL, Pfaffenbach D, Davis MD. Cell turnover of capillaries. Lab Invest 1967; 17: 738-43.
6. Freyer JP, Tustanoff E, Franko AJ, Sutherland RM. In situ oxygen consumption rates of cells in V-79 multicellular spheroids during growth. J Cell Physiol 1984; 118: 53-61.
7. Butler LM, Agus DB, Scher HI, et al. Suberoylanilide hydroxamic acid, an inhibitor of histone deacetylase, suppresses the growth of prostate cancer cells in vitro and in vivo. Cancer Res 2000; 60: 5165-70.
8. Byers HR, Etoh T, Doherty JR, Sober AJ, Mihm MC, Jr. Cell migration and actin organization in cultured human primary, recurrent cutaneous and metastatic melanoma. Time-lapse and image analysis. Am J Pathol 1991; 139: 423-35.
9. Zaman MH, Trapani LM, Siemeski A, et al. Migration of tumor cells in 3D matrices is governed by matrix stiffness along with cell-matrix adhesion and proteolysis (vol 103, pg 10889, 2006). P Natl Acad Sci USA 2006; 103: 13897-.
10. Anderson ARA. A hybrid mathematical model of solid tumour invasion: the importance of cell adhesion. Math Med Biol 2005; 22: 163-86.
11. Tsai AG, Friesenecker B, Mazzoni MC, et al. Microvascular and tissue oxygen gradients in the rat mesentery. P Natl Acad Sci USA 1998; 95: 6590-5.
12. Olive PL, Vikse C, Trotter MJ. Measurement of oxygen diffusion distance in tumor cubes using a fluorescent hypoxia probe. Int J Radiat Oncol Biol Phys 1992; 22: 397-402.
